# Insomnia severity and its relationship with demographics, pain features, anxiety, and depression in older adults with and without pain: cross-sectional population-based results from the PainS65+ cohort

**DOI:** 10.1186/s12991-017-0137-3

**Published:** 2017-02-23

**Authors:** Elena Dragioti, Lars-Åke Levin, Lars Bernfort, Britt Larsson, Björn Gerdle

**Affiliations:** 1 Pain and Rehabilitation Centre, Department of Medical and Health Sciences (IMH), Linköping University, 581 85 Linköping, Sweden; 2 Division of Health Care Analysis, Department of Medical and Health Sciences, Linköping University, 581 85 Linköping, Sweden

**Keywords:** Pain, Chronic, Anxiety, Depression, Elderly, Insomnia, Insomnia Severity Index

## Abstract

**Background:**

Insomnia is a major cause of concern in the elderly with and without pain. This study set out to examine the insomnia and its correlates in a large sample of community adults aged ≥65 years.

**Methods:**

A cross-sectional postal survey was completed by 6205 older individuals (53.8% women; mean age = 76.2 years; SD = 7.5). The participants also completed the Insomnia Severity Index (ISI) and questionnaires assessing pain intensity, pain spreading, anxiety, depression, and basic demographic information. The sample was divided into three groups based on the presence and duration of pain: chronic pain (CP; *n* = 2790), subacute pain (SP; *n* = 510), and no pain (NP; *n* = 2905).

**Results:**

A proportion of each of the groups had an ISI score of 15 or greater (i.e., clinical insomnia): CP = 24.6%; SP = 21.3%; and NP = 13.0%. The average scores of ISI differed significantly among CP, SP, and NP groups (*p* < 0.001). Stratified regression analyses showed that pain intensity, pain spreading, anxiety, and depression were independently related to insomnia in the CP group. Anxiety and depression were independently related to insomnia in the SP group, but only anxiety was significantly associated with insomnia in the NP group. Age and sex were not associated with insomnia.

**Conclusions:**

This study confirms that insomnia is not associated with chronological aging per se within the elderly population. Although the possible associations of insomnia with pain are complex, ensuing from pain intensity, pain spreading, anxiety, and depression, our results highlighted that anxiety was more strongly associated with insomnia in all groups than the depression and pain characteristics. Therapeutic plans should consider these relations during the course of pain, and a comprehensive assessment including both pain and psychological features is essential when older people are seeking primary health care for insomnia complaints.

## Background

One of the most common sleep disturbances among older adults is insomnia [1–5]. Insomnia is defined as a “complaint of insufficient and non-restorative sleep described by the inability to initiate and/or maintain sleep” [6]. In older adults, the overall prevalence of insomnia ranges from 30 to 48% [7, 8] with an annual incidence of near 5% [9]. Furthermore, the prevalence of clinical insomnia in this population is estimated to be over 20% [10], and the incidence of insomnia symptoms is expected to be even higher among men 85 years and older [9].

The impact of insomnia is particularly high in the elderly [2, 11, 14–26]. Insomnia incurs substantial adverse consequences for the individual and for society [8]. Hence, insomnia in older adults is associated with decreased quality of life [11, 12], impaired concentration and memory [13], cognitive decline [14–16], increased incidence of medical and psychiatric disorders [8, 17–19], and increased risk of falls, fractures, and mortality [20–22]. Moreover, large societal costs and increased health care resource use have been noted [8, 23]. For example, an overview study reported that the total direct, indirect, and related annual costs of insomnia in United States were estimated to be between 30 and 35 billion US dollars [23].

Traditionally, it has been argued that certain alterations in the circadian rhythm are connected to insomnia as people age [19]. The most significant demographic predictors of insomnia are older age and female sex [7, 9, 17, 24, 25]. However, insomnia in older adults is probably the result of morbidity rather than aging per se [26–28]. Thus, in the majority of older adults, insomnia is believed to be due to medical disorders (especially chronic pain and respiratory and neurological disorders), psychiatric disorders (especially depression and/or anxiety), and medications (especially anticholinergics and antidepressants) [8, 17, 19, 26, 27, 29, 30]. Older adults with chronic pain seem to be a group with a high risk of being afflicted by insomnia [27, 31, 32] with a prevalence of various sleep problems including insomnia ranging from 13 to 62% [32]. In addition, impaired sleep quality is associated with higher pain intensity [33, 34]. In this vein, a recent article has suggested that insomnia in older adults should be considered a “multifactorial geriatric syndrome” [35]. Because sleep complaints are influenced by a combination of medical, physical, cognitive, psychological, and social issues, diagnostic assessments and treatment plans should take into account all these issues rather than considering insomnia as an inevitable consequence of aging [35].

Considering all of this evidence, it is imperative that investigations of insomnia in older adults should account for level of pain. Although extensive research has been conducted on the relationship between insomnia and pain in older adults, to the best of our knowledge, no study has examined insomnia severity with respect to different pain groups or duration and course of pain. In addition, these studies have produced contradicting results as the definition of insomnia varies between studies [7, 36, 37]. Some studies have also emphasized that emotional symptoms like depression and anxiety have a stronger association with insomnia in pain populations than the psychical symptoms like the pain itself [34, 38–40]. However, little is known about the relationship between depression, anxiety, and pain symptoms that contribute to insomnia in the elderly people. Hence, this study evaluates the severity of insomnia and its relationship with, age, sex, pain intensity, pain spreading, anxiety, and depression in a large-scale population-based study in older adults with pain (i.e., chronic and subacute pain) and without pain.

## Methods

This cross-sectional study used data from the PainS65+ cohort [41] and based on a sampling frame from the Swedish Total Population Register (TPR). From this registry, 10,000 subjects aged 65 years and older, were randomly selected for the following age strata: 65–69; 70–74; 75–79; 80–84; and 85 years and older. The data were collected during 1 year period (from October 2012 to January 2013) using a postal survey, as a part of a larger project in which the health status of the elderly individuals in the general population was investigated in various domains [41, 42]. The parts of the instrument used in this study are described below. An overview of all parts of the survey and the details of the subsequent procedure are described elsewhere [41, 42]. The study was approved by the Regional Ethics Research Committee in Linköping, Sweden (Dnr: 2012/154-31).

### Measurements

#### Insomnia

Insomnia was assessed using the Insomnia Severity Index (ISI), a reliable and valid instrument with excellent internal consistency [43]. The ISI is a seven-item self-report instrument that assesses the nature, severity, and impact of insomnia [43, 44] using a five-point Likert scale (0 = no problem and 4 = very severe problem), yielding a total score ranging between 0 and 28. A higher score indicates greater insomnia severity. The total score is further divided into four categories: no clinically significant insomnia (ISI = 0–7); sub-threshold insomnia (ISI = 8–14); moderate clinical insomnia (ISI = 15–21); and severe clinical insomnia (ISI = 22–28). Clinical insomnia was defined as the sum of moderate clinical insomnia and severe clinical insomnia [39, 40]. This study presents the ISI total score as well as the subcategory scores.

#### Definition of pain categories

The pain was defined by a single question and one follow-up question with respect to the presence and time course of pain: “Do you usually have pain?” (yes/no) and “If yes, has your pain lasted fewer than 3 months or more than 3 months?” The subjects who responded “no” were assigned to the no pain (NP) group. Subjects who responded “yes, with more than 3 months” were assigned to the chronic pain (CP) group. Accordingly, the subjects who responded “yes, with fewer than 3 months” were assigned to the subacute pain (SP) group.

#### Pain intensity

The average pain intensity for the previous 7 days was registered using an 11-point numeric rating scale (NRS7d) with the anchors 0 (no pain) and 10 (worst imaginable pain) [45]. The subjects in the NP group marked 0 in this scale.

#### Pain spreading

Pain spreading (i.e., spatial extent) was assessed by having the respondents’ mark where they experience pain on a body manikin with 45 predefined anatomical areas. The subjects marked the anatomical areas where they experienced either subacute or chronic pain during the previous 7 days [46]. The subjects in the NP group did not mark any anatomical areas. Thus, the number of sites associated with pain ranged between 0 and 45; higher values indicated higher pain spreading.

#### Anxiety and depression

Two subscales of the General Well-Being Schedule (GWBS) were used to assess anxiety (items 2, 5, 8, 16) and depression (items 4, 12, 18): GWBS-anxiety (range 0–25) and GWBS-depression (range 0–20). The GWBS is a common instrument for assessing life satisfaction and level of psychological distress [47]. It consists of 18 items yielding a total score ranging from 0 to 110 (high score indicating positive well-being and low distress). The first 14 questions use a six-point rating scale (anchors 0 and 5) that represents either intensity or frequency, and the remaining four items use an 11-point rating scale with the anchors 0 (very concerned) and 10 (not concerned). The GWBS has good internal consistency, test–retest reliability, and validity [47]. Four subscales of the instrument are not used in this study: GWBS-positive well-being: items 1, 6, 11; range 0–15; GWBS-self-control: items 3, 7, 13; range 0–15; GWBS-vitality: items 9, 14, 17; range 0–20; and GWBS-general health: items 10, 15; range 0–15 [48].

#### Data analysis

The statistics were performed using the statistical package IBM SPSS Statistics (version 23.0; IBM Inc., New York, USA). In all tests, a *p* value of ≤0.05 (two-tailed) was considered significant. Continuous data are reported as the mean and standard deviation (SD), and the categorical data are represented as n (%). Both analysis of variance (ANOVA) and Student’s independent *t* test were used for the continuous variables and Chi-square *t* tests were used for the categorical variables. Post hoc comparisons (Bonferroni criterion used for the total score of ISI and Dennett’s test used for the subcategories of ISI) were also performed when significant differences (*p* < 0.05) in ANOVA tests were identified. Pearson correlation analysis was used for bivariate correlations (i.e., investigating the correlations between total score of ISI and the other examined variables). Multiple linear regression (MLR) analyses were performed to regress insomnia (a dependent variable treated as a continuous variable) using age, female sex, pain intensity, pain spreading, and anxiety and depression (exploratory variables) with each pain group. Multicollinearity was assessed by examining tolerance and the variance inflation factor (VIF). A tolerance of less than 0.20 or 0.10 and/or a VIF of 5 or 10 and above indicate multicollinearity [49].

In addition, ordinal logistic regression (OLR) models were used with the ISI categories and these categories were treated as ordinal outcome variables. Odds ratios (OR) and 95% confidence intervals (CI) are reported. All analyses were stratified by the three pain groups.

## Results

### Sample characteristics

The CP group included 2790 individuals (61.1% women; average age 76.2 years [SD 7.5]), the SP group included 510 individuals (54.1% women; average age 75.6 years [SD 6.9]), and the NP group included 2905 individuals (46.1% women; average age 75.9 years [SD 7.6]). Age distribution did not differ among the three pain groups (*p* = 0.47), but there was a significant difference in gender distribution (*p* < 0.001). There were more women than men in the two pain groups (CP group: 61.1 vs 38.9% and SP group: 54.1 vs 45.9%) and more men than women in the NP group (53.9 vs 46.1%). Details of the socio-demographic characteristics and response rates of the total sample are described elsewhere [42].

### Insomnia severity and its relationship with other variables in the total sample

**Fig. 1:**
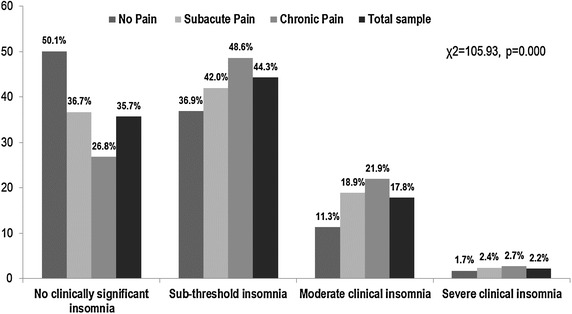
Prevalence of insomnia severity in older adults with and without pain. The *x axis* represents the proportions of the study sample %; the *y axis* represents the categories of insomnia by the three pain groups and the total sample

**Fig. 2:**
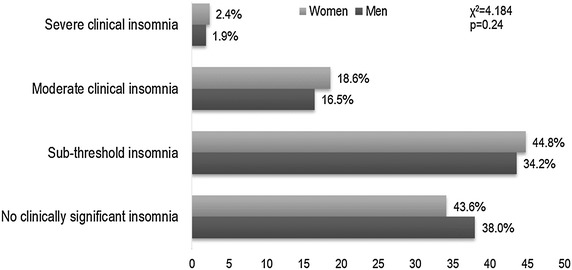
Prevalence of insomnia severity between the two sexes. The *x axis* represents the categories of insomnia by sex; the *y axis* represents the proportions of the study sample %

The average ISI total score was 9.8 ± 5.5. The distribution in the different categories of ISI showed that 35.7% had no clinically significant insomnia, 44.3% had sub-threshold insomnia, 17.8% had moderate clinical insomnia, and 2.2% had severe clinical insomnia (Fig. 1). That is, 20% of the total sample had clinical insomnia (ISI ≥ 15). A significant difference in the total score of ISI existed between the two sexes (men 9.4 ± 5.5; women 10.1 ± 5.5; *p* = 0.003), but this difference was not confirmed when we compared the distribution of the four categories of ISI between the two sexes (Fig. 2).

The four categories of ISI with respect to the other investigated variables are presented in Table 1, part a. Prominent and significant differences were found for all investigated variables except for age. More specifically, higher pain intensity, pain spreading, anxiety, and depression were more common in severe clinical insomnia (ISI ≥ 22) compared to no clinically significant insomnia.

**Table 1:** Insomnia severity in individuals with and without pain and the categories of insomnia related to age, pain intensity, pain spreading, anxiety, and depression

Subgroup of ISI	All	No clinically significant insomnia (NCSI)	Sub-threshold insomnia (S-TI)	Moderate clinical insomnia (MCI)	Severe clinical insomnia (SCl)	Statistics (ANOVA)	Post hoc comparisons (Dunnett t)^a^		
Variables	Mean ± SD	Mean ± SD	Mean ± SD	Mean ± SD	Mean ± SD	*p* value	S-TI vs NCSI	MCI vs NCSI	SCl vs NCSI
a. Total sample (*n* = 6205)									
Age (years)	76.3 ± 7.4	76.5 ± 7.2	76.0 ± 7.4	76.5 ± 7.6	78.1 ± 8.3	0.175	NA	NA	NA
Pain intensity	4.2 ± 2.6	3.2 ± 2.6	4.5 ± 2.4	5.3 ± 2.5	6.0 ± 2.7	<0.001	***	***	***
Pain spreading	5.6 ± 5.4	4.4 ± 4.6	5.3 ± 4.7	7.7 ± 7.1	7.4 ± 7.1	<0.001	*	***	***
GWBS-anxiety	6.4 ± 4.5	4.1 ± 3.7	6.6 ± 4.3	9.7 ± 5.2	12.8 ± 5.9	<0.001	***	***	***
GWBS-depression	5.8 ± 3.8	4.1 ± 3.2	5.9 ± 3.3	8.2 ± 3.9	10.5 ± 4.8	<0.001	***	***	***
b. Chronic pain (*n* = 2790)									
Age (years)	76.2 ± 7.5	76.6 ± 7.6	75.6 ± 7.2	76.9 ± 7.8	78.7 ± 7.6	0.015	ns	ns	**
Pain intensity	5.1 ± 2.1	4.4 ± 2.1	5.1 ± 1.9	5.7 ± 2.2	6.5 ± 2.48	<0.001	***	***	***
Pain spreading	6.2 ± 5.6	5.1 ± 4.9	5.8 ± 4.8	8.3 ± 7.2	8.0 ± 7.2	<0.001	ns	***	***
GWBS-anxiety	7.4 ± 4.9	5.0 ± 4.1	7.2 ± 4.3	10.0 ± 5.1	13.5 ± 6.7	<0.001	***	***	***
GWBS-depression	6.3 ± 3.8	4.8 ± 3.4	6.1 ± 3.3	8.2 ± 3.8	10.7 ± 5.1	<0.001	***	***	***
c. Subacute pain (*n* = 510)									
Age (years)	75.6 ± 6.9	75.2 ± 6.9	75.4 ± 6.8	76.3 ± 7.3	76.0 ± 6.9	0.890	NA	NA	NA
Pain intensity	4.4 ± 1.8	4.1 ± 1.6	4.4 ± 1.9	4.8 ± 1.8	5.5 ± 0.7	0.304	NA	NA	NA
Pain spreading	3.1 ± 2.1	2.9 ± 2.1	3.1 ± 2.2	3.4 ± 2.2	2.0 ± 0.0	0.683	NA	NA	NA
GWBS-anxiety	6.5 ± 4.8	4.4 ± 3.5	6.6 ± 4.4	10.0 ± 5.3	13.7 ± 2.5	<0.001	**	***	***
GWBS-depression	5.8 ± 3.7	4.3 ± 3.1	5.7 ± 3.4	8.5 ± 3.7	10.0 ± 3.6	<0.001	*	***	***
d. No pain (*n* = 2905)									
Age (years)	75.9 ± 7.6	76.5 ± 6.9	76.4 ± 7.8	75.6 ± 7.4	77.1 ± 10.7	0.911	NA	NA	NA
Pain intensity	0.0 ± 0.0	0.0 ± 0.0	0.0 ± 0.0	0.0 ± 0.0	0.0 ± 0.0	NA	NA	NA	NA
Pain spreading	0.0 ± 0.0	0.0 ± 0.0	0.0 ± 0.0	0.0 ± 0.0	0.0 ± 0.0	NA	NA	NA	NA
GWBS-anxiety	4.8 ± 4.2	3.3 ± 3.3	5.3 ± 4.1	8.6 ± 4.8	10.9 ± 4.3	<0.001	***	***	***
GWBS-depression	4.7 ± 3.6	3.4 ± 2.9	5.2 ± 3.3	7.7 ± 4.2	9.9 ± 4.2	<0.001	***	***	***

*NCSI* no clinically significant insomnia, *S-TI* sub-threshold insomnia, *MCI* moderate clinical insomnia, *SCl* severe clinical insomnia, *ISI* Insomnia Severity Index, *GWBS* general well-being schedule, *NA* not applicable

^a^Dunnett *t* tests treat one group as a control (No clinically significant insomnia as control group), and compare all other groups against it; *ns* non-significant at *p* < 0.05; *NA* not applicable

* *p* < 0.05, ** *p* < 0.01, *** *p* < 0.001

### Insomnia severity in the chronic pain group and comparisons with subacute pain and no pain group

**Fig. 3:**
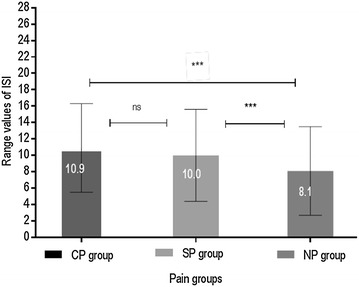
Distribution and comparisons of mean values of ISI among older adults with chronic pain, subacute pain, and no pain. *ISI* Insomnia Severity Index, *CP* chronic pain, *SP* subacute pain, *NP* no pain; *denotes significant differences between the pain groups (***p < 0.001) and ns denotes non-significant differences at *p* < 0.05

The average score of ISI in individuals with CP was 10.9 ± 5.4. The average scores of ISI differed very clearly between CP, SP, and NP groups (*p* < 0.001) (Fig. 3). Post hoc comparisons found that the average ISI in the NP group was significantly lower compared to the SP and CP groups (*p* < 0.001). No significant differences between CP and SP groups were found (*p* = 0.12).

The distribution in the different ISI categories showed that in the CP group 26.8% had no clinically significant insomnia, 48.6% had sub-threshold insomnia, 21.9% had moderate clinical insomnia, and 2.7% had severe clinical insomnia. That is, about 25.0% of older individuals with chronic pain had clinical insomnia (ISI ≥ 15). The overall fraction of clinical insomnia was 21.3% in SP group and 13.0% in NP group (Fig. 1). Within the CP group, a significant difference in the total score of ISI between the two sexes was found (men 10.4 ± 5.3; women 11.1 ± 5.4; *p* = 0.03), but this was not the case for SP (*p* = 0.86) and NP groups (*p* = 0.56).

### Comparisons of ISI categories with the examined variables stratified by the three pain groups

The four ISI categories with respect to the other investigated variables and the three pain groups are presented in Table 1, parts b–d. In the CP group, prominent and significant differences emerged for all investigated variables. In the SP group and NP group, significant differences were found between ISI subgroups and anxiety and depression. In general, severe clinical insomnia (ISI ≥ 22) was associated with the worst situation with respect to the examined variables compared to no clinically significant insomnia.

### Associations of ISI total scores and ISI categories with the examined variables stratified by the three pain groups

Correlation and multiple linear regression (MLR) analyses were conducted to examine the relationship between ISI total score and the other possible regressor variables; Table 2 summarizes the stratified correlations and the MLR analysis results by each pain group. Multicollinearity was rejected: the average tolerance was 0.70 and the average VIF was 1.42 for all independent variables.

**Table 2:** Correlations among variables and results from the linear regression models of ISI total score (continue variable) stratified by the three pain groups

Variable	Correlations with ISI (*r*)	Multiple regression weights		*R* ^2^
		*Β*	*β*	
a. Model 1 Chronic pain (*n* = 2790)				0.24
Age (years)	0.43	−0.017	−0.023	
Female sex	0.19	0.235	0. 021	
Pain intensity	0.27***	0.356***	0.138***	
Pain spreading	0.20***	0.069*	0.075*	
GWBS-anxiety	0.43***	0.259***	0.237***	
GWBS-depression	0.40***	0.261***	0.132***	
b. Model 2 Subacute pain (*n* = 510)				0.26
Age (years)	0.01	−0.039	−0.053	
Female sex	0.07	0.188	0.018	
Pain intensity	0.16*	0.246	0.086	
Pain spreading	0.06	0.010	0.004	
GWBS-anxiety	0.48***	0.294*	0.274*	
GWBS-depression	0.46***	0.344*	0.246*	
c. Model 3 No pain (*n* = 2905)				0.44
Age (years)	0.21	0.045	0.056	
Female sex	−0.05	−0.061	−0.057	
Pain intensity	NA	NA	NA	
Pain spreading	NA	NA	NA	
GWBS-anxiety	0.66***	0.747***	0.581***	
GWBS-depression	0.53***	0.136	0.099	

*ISI* Insomnia Severity Index, *GWBS* general well-being schedule, *r* correlation coefficient, *B* unstandardized regression coefficients, *β* standardized regression coefficients, *R* ^*2*^ multiple correlation coefficient squared, *NA* not applicable

* *p* < 0.05, ** *p* < 0.01, *** *p* < 0.001

In the CP group, both correlation and MLR analyses showed that pain intensity, pain spreading, anxiety, and depression were positively and significantly correlated with the ISI scores (Table 2; Model 1). Hence, older individuals with CP and higher scores on these variables were expected to have higher ISI scores. Anxiety was more important as a regressor than pain intensity, pain spreading, and depression. The MLR model with all six regressors explained 24% of the total variance [*R* ^2^ = 0.24, *F* (6337.8) = 57.81, *p* < 0.001]. Age and female sex did not contribute significantly to the model. In the SP group, anxiety and depression had a clear association with ISI scores, but age, female sex, pain intensity, and pain spreading did not significantly contribute to the model. The MLR model with all six regressors explained 26% of the total variance [*R* ^2^ = 0.26, *F* (923.1) = 9.21, *p* < 0.001] (Table 2; Model 2). In the NP group, only anxiety was significantly associated with the ISI scores. In this model, both pain variables (i.e., pain intensity and spreading) were not entered in the model because the mean score on those variables was zero. The MLR model with the four relevant regressors explained 44% of the total variance [*R* ^2^ = 0.44, *F* (601.6) = 11.61, *p* < 0.001] (Table 2; Model 3). After we regressed the ISI categories (no clinically significant insomnia vs. sub-threshold insomnia, moderate insomnia, and severe clinical insomnia) as ordinal outcomes, we found identical results with respect to the most important variables (Table 3; Models 1–3).

**Table 3:** Results of ordinal logistic regression models of categories of ISI treated as an ordinal outcome stratified by the three pain groups

Variable	Μultiple regression weights		
	Wald	OR (95% CI)	Nagelkerke *R* ^2^
a. Model 1 Chronic pain (*n* = 2790)			0.22
Age (years)	1.013	0.99 (0.97–1.01)	
Female sex	1.116	1.03 (0.80–1.33)	
Pain intensity	15.501	1.13 (1.06–1.20)***	
Pain spreading	8.930	1.04 (1.01–1.06)**	
GWBS-anxiety	29.452	1.11 (1.06–1.15)***	
GWBS-depression	11.475	1.09 (1.03–1.14)***	
b. Model 2 Subacute pain (*n* = 510)			0.23
Age (years)	1.189	0.98 (0.93–1.02)	
Female sex	0.398	0.99 (0.51–1.57)	
Pain intensity	0.979	1.10 (0.91–1.34)	
Pain spreading	0.052	1.01 (0.97–1.15)	
GWBS-anxiety	6.547	1.15 (1.03–1.28)*	
GWBS-depression	3.398	1.08 (1.01-1.25)*	
c. Model 3 No pain (n = 2905)			0.41
Age (years)	0.057	0.99 (0.96–1.01)	
Female sex	0.187	0.99 (0.76–1.43)	
Pain intensity	NA	NA	
Pain spreading	NA	NA	
GWBS-anxiety	6.059	1.13 (1.06–1.18)*	
GWBS-depression	0.594	1.10 (0.99–1.24)	

*ISI* Insomnia Severity Index, *GWBS* general well-being schedule, *OR* odds ratio, *R* ^*2*^ multiple correlation coefficient squared, *NA* not applicable

* *p* < 0.05, ** *p* < 0.01, *** *p* < 0.001

## Discussion

The main findings of this study were as follows:

- The overall prevalence of clinical insomnia in the total sample was 20.0%. Older adults with CP had the highest prevalence of clinical insomnia (24.6%).
- Age and sex were not associated with either total ISI score or ISI categories, regardless of pain group.
- Higher pain intensity, pain spreading, anxiety, and depression were more common in severe insomnia, moderate clinical insomnia, and sub-threshold insomnia compared to no clinically significant insomnia.
- The multivariate stratified analyses revealed the following associations: pain intensity, pain spreading, anxiety, and depression were independently related to insomnia in the CP group. Anxiety and depression were independently related to insomnia in the SP group, but only anxiety was significantly associated with insomnia in the NP group.

Overall, 20% of the participants reported clinical insomnia (ISI ≥ 15) [44], a rate lower compared to that reported in most other studies of elderly populations [1, 4, 11, 50]. Foley et al., for example, in a large sample of 9000 participants aged 65 years and older found that the estimated insomnia symptoms ranged between 23 and 34% [4]. Our study found a higher prevalence of insomnia than the findings of an investigation of 47,700 individuals in Norway (i.e., a similar geo-cultural environment to Sweden) where the prevalence rate of insomnia symptoms was 13.5% [17]. That study, however, did not report results specifically for the elderly. Consistent with previous reports [24–27, 29, 30], we also found that the respondents in CP reported higher prevalence (24.6%) of clinical insomnia than respondents in the SP and NP groups. Indeed, there is evidence that patients with CP tended to demonstrate more sleep fragmentation, longer sleep latency, lower sleep quality, and shorter sleep duration [54]. Explanations for the observed differences of prevalence estimates may include the great variability of measurements and definitions of insomnia [7, 36, 51]. Studies using DSM-5 [52] criteria for insomnia reveal lower prevalence of insomnia in the elderly (4–12%). This discrepancy may also be the result of the age range of the studied population, pain definitions, and socioeconomic and cultural differences of the participants [7, 17, 24, 35].

In contradiction to some previous research findings [4, 9, 17, 24, 25], age and sex were not associated with insomnia symptoms or clinical insomnia. Although we found that in the CP group severe clinical insomnia compared to no clinically significant insomnia was more common in the older ages (Table 1, part b), this difference was not confirmed in the regression analysis. Nevertheless, our results are similar to some prospective studies that found no age effect related to insomnia [53, 54]. Moreover, our results (Tables 2, 3) suggest that insomnia cannot be explained by the chronologically age-related changes in the elderly population, but rather is explained by aging-related changes due to various mental and physical morbidities including pain [10, 11, 21, 27, 28, 30, 31, 48]. Therefore, as recently suggested, insomnia should be considered a “multifactorial geriatric syndrome” [32]. Similarly, we found that the total scores of insomnia were higher in women compared to men in the whole sample and in the CP group, but these sex differences were not confirmed in the regression analyses (Tables 2, 3). This finding is consistent with the suggestion that the relationship between female sex and insomnia (especially in the elderly) may be explained by an array of other factors such as depression, chronic diseases, living alone, marital status, occupational status, and social support deficits [35, 55].

The stratified multivariate analysis of the three pain groups revealed that in the CP group higher levels of pain intensity, pain spreading, anxiety, and depression had direct links to higher severity of insomnia. The most important regressors for both total ISI score and clinical insomnia were pain intensity and anxiety. This result is somewhat consistent with previous reports [24, 33, 34, 38, 56]. Our results, however, are not in line with some studies that report that insomnia in CP is more strongly related to depression and low mood than to pain intensity [38, 39], but in line with studies reporting that anxiety is more strongly related to insomnia than to pain intensity [34, 40]. Generally, the results of the regressions are consistent with previous reports that found strong relationships between both pain and psychological strain and the variability of sleep duration and fragmentation in various populations, including older adults [17, 24, 29, 34, 57].

In the SP group, both anxiety and depression had clear positive associations with total ISI score and clinically significant insomnia, whereas in the NP group only anxiety was significantly associated with insomnia. Hence, in these two groups, anxiety also had a stronger relationship with insomnia than depression, a finding that is not substantiated in previous studies [4, 26, 38, 39, 58]. One possible explanation for the stronger impact of anxiety in the present population could be the fact that in elderly depression symptoms are conflated with somatic complaints, and probably those symptoms are being underestimated and unreported by the respondents [59]. On the other hand, anxiety may more likely be the result of stressful events associated with aging, and perhaps this anxiety increases the likelihood of insomnia via increased levels of arousal [60]. It is also possible that anxiety and depression are interrelated with insomnia through different paths. According to previous prospective research, anxiety may more likely act as a pathway to insomnia while depression may more likely act as a consequence of insomnia [61, 62]. In any case, the relationship between insomnia and pain as well as other morbidities is difficult to interpret [27, 31, 32] and more studies, especially longitudinal studies, concerning the nature and the direction of these associations are needed.

To our best knowledge, this is the first study to evaluate insomnia severity in a large random sample of older adults with and without pain. This study’s limitations include the issues associated with collecting data via a self-reported instrument and the inherent limitations of a cross-sectional study design. In addition, this study evaluated a limited set of variables. We did not examine the role of other factors such as medications, especially hypnotics or other physical comorbidities; life-style factors such as nicotine, caffeine, and alcohol use; or stressful life traumatic events that might be independently related to insomnia [25].

## Conclusions

In conclusion, this study suggests that the relationship between insomnia severity and chronic pain in older adults is very complex, ensuing from pain intensity, pain spreading, anxiety, and depression. In subacute pain, pain symptoms seem to have no effect while a predominance of a psychological strain may account for higher levels of insomnia and clinical insomnia. Conversely, in older individuals without pain, anxiety had a clear positive relationship to both insomnia and clinical insomnia. Taken together, these results indicate that ongoing specific attention from health care providers on this topic is required to ensure that the best possible insomnia treatment modalities are made available to the elderly. For example, approaches including both psychological and pain management components would be beneficial for individuals with CP, whereas psychological approaches targeting depression and/or anxiety might be suitable for individuals who experience short durations of pain or no pain. This study also suggests that a comprehensive assessment including both pain and psychological aspects are essential when older people are seeking primary health care for insomnia complaints. Further research is also needed that defines these multifactorial relations so that elderly patients suffering from insomnia receive the most effective insomnia treatments regardless of their pain level.

## Abbreviations

ANOVA: one way analysis of variance
CP: chronic pain
GWBS: the general well-being schedule
ISI: Insomnia Severity Index
MLR: multiple linear regression
NP: no pain
NRS7d: numeric rating scale for the previous 7 days
OLR: ordinal logistic regression
SD: standard deviation
SP: subacute pain
TPR: total population register
